# Nozzle Wear in Abrasive Water Jet Based on Numerical Simulation

**DOI:** 10.3390/ma17143585

**Published:** 2024-07-19

**Authors:** Xuhong Chen, Hongji Yu, Haihong Pan, Lin Chen, Hui You, Xubin Liang

**Affiliations:** Precision Polishing and Measuring Laboratory, Guangxi University, Nanning 530000, China; 18172423561@163.com (X.C.); yhj950905@163.com (H.Y.); gxdxcl@163.com (L.C.); hyou@gxu.edu.cn (H.Y.); gxnnlxbwlh@163.com (X.L.)

**Keywords:** nozzle wear, numerical simulation, particle diameter, erosion rate, Euler-Lagrange methodology, numerical simulation

## Abstract

Particle diameters and jet pressure in abrasive water jet (AWJ) are significant jet properties which deserve a better understanding for improving AWJ machining performance. Some influence factors have been verified regarding nozzle wear in abrasive water jet polishing application. A three-dimensional model of a nozzle is established to analyze the influence of internal multi-phase flow field distribution, which is based on Euler-Lagrange methodology. With the increase of jet pressure, the erosion rate decreases; with the increase of the diameter and mass flow rate of the erosion particles, the erosion speed increases as well. When the diameter of the outlet is worn to 1.6 mm, the pressure on the work piece caused by the abrasive water jet increases by more than double compared to the non-worn nozzle; when the diameter of the nozzle outlet is worn to 1.6 mm, the shear force is 2.5 times higher than the shear force when the diameter is 1.0, which means that the jet force is divergent when the diameter is 1.6 mm, and the damage of the work piece is very serious. The obtained results could improve polishing efficiency on the work piece, extend nozzle lifetimes, and guide the future design of AWJ nozzles.

## 1. Introduction

Abrasive water jet technology is an improved method of pure water jet technology, specifically referring to abrasive water mixed with abrasive particles which are sprayed out at high speed through a nozzle and act on the surface of a work piece. The erosion and shear effects generated by the high-speed movement of abrasive particles in the jet can remove materials and achieve the purpose of polishing the work piece. Compared to other, traditional machining technologies, abrasive water jet polishing technology has higher flexibility and better adaptability. The abrasive flow of the polishing system can complete special structure, such as polishing an cavity of a hole. Therefore, the application range of abrasive water jet polishing technology is wider, and it has been well applied in scenarios involving surface treatment, mechanical manufacturing, and other fields [1]. Madhusarathi et al. [2] studied the effects of abrasive water jet system parameters and jet nozzle geometry on nozzle wear. Konstantin et al. [3] studied the effect of water inlet pressure on nozzle wear through experiments, obtained the limit value of nozzle wear within a certain working time, and established a generalized equation for jet nozzle wear. Kamarudin et al. [4] used computational fluid dynamics (CFD) software, Fluent 6.3.26, to optimize and predict the abrasive water jet model, and numerically simulated several grid partitioning methods for abrasive water jet erosion. The calculation time and accuracy of each method were discussed, and the results showed that the tetrahedral grid partitioning method required less time for numerical simulation of the abrasive water jet. Gabriele et al. [5] proposed a numerical method for studying the erosion phenomenon inside abrasive water jet nozzles. By combining the multi-scale algorithm of CFD-DEM with the erosion model, they captured the erosion effect of abrasives on the inner wall of the nozzle due to the cumulative impact phenomenon. Wu et al. [6] studied three parameters that influence solid acceleration and nozzle wear in detail: focusing tube converging angle, particle inlet position and particle inlet angle. Syazwani et al. [7] discussed a review on nozzle wear factors including nozzle length, nozzle inlet angle, nozzle diameter, orifice diameter, abrasive flow rate and water pressure in abrasive water jet machining. Perec et al. [8] calculated the mass loss of the focusing tube by different sizes of garnet striking in the cavity of the focusing tube and estimated the service life of the focusing tube. Mostofa et al. [9] studied the flow characteristics of the AWJ on the inner surface of the nozzle and the effect of the water jet on the wear rate of the nozzle surface. Zou et al. [10] studied the multiphase flow of abrasive particles by using the Euler-Lagrange method, which considers the shape factor of the particle and defines an effective model for the particle-to-wall wear model. Excessive jet velocity and jet impact force can have a negative impact on the surface polishing accuracy on the work piece, leaving obvious grooves and corrosion points, leading to poor surface quality of the work piece [11]. Chen et al. [12] show that the wear of the nozzle will be greatly affected when the length of the nozzle is changed in the range of the severe wear area. Du et al. [13] study the influences of cylinder length and inlet angle on wear behavior of pre-mixed AWJ nozzles by wear tests and wear simulations. The Widosinski curve of the optimal section nozzle improves by 5.64% and the lifecycle increases by 43.2% compared with the commercial production single cone nozzle [14]. The experimental results have shown that laminated ceramic nozzles have superior erosion wear resistance to that of the homologous stress-free nozzles [15]. Experiments and simulations have shown that an incidence angle of the dual gradient nozzle greater than 30 can reduce nozzle wear issues [6]. Traverse speed and nozzle diameter are significant parameters in the process of abrasive water jet cutting [16].

The goal of this study is the development and validation of an internal three-phase flow model of the abrasive water jet with the capability of predicting the acceleration of solid particles and the wear of the nozzle wall. The research content of this paper mainly includes:

1. A brief theoretical analysis of the fluid movement in the internal flow field of the nozzle using fluid dynamics, providing a theoretical basis for the process of numerical simulation.
2. Using Fluent 6.3.26, a discrete phase model was used to analyze the erosion of the abrasive on the inner cavity of the jet polishing nozzle, and the effects of three parameters on the maximum erosion rate of the nozzle were analyzed and compared by changing the abrasive diameter, inlet pressure, and abrasive flow rate.
3. Analysis of the simulation results of abrasive erosion on the inner cavity of the jet polishing nozzle and the prediction of the changes in the structure of the nozzle’s inner cavity based on existing data and references.
4. Use of CFD to model and simulate the inner cavity of the nozzle before and after wear and compare the impact of structural changes on the nozzle jet and the impact of the jet on the target work piece after structural changes.

## 2. Erosion Mechanism

### 2.1. Numerical Simulation Calculation Model

The turbulence numerical simulation method used in this article adopts the turbulent viscosity method in the Reynolds mean time equation method. To close the average Reynolds equation of turbulence, various turbulence mathematical models have been established, among which the two-equation model in the turbulence viscosity method is widely used for turbulence numerical simulation, including the Standard k-ε Model, RNG k-ε Model and Realizable k-ε Model [3].

**Figure 1:**
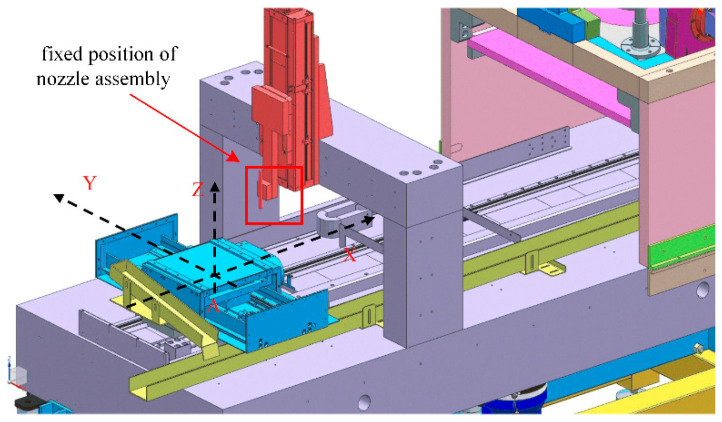
Three-dimensional model of AWJ platform.

**Figure 2:**
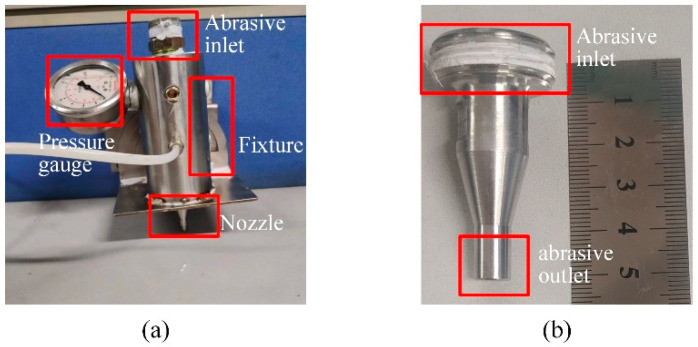
Physical diagram of nozzle assembly. (**a**) Physical image of spray assembly parts; (**b**) enlarged view of nozzle in the figure (**a**).

The results of nozzle numerical calculations are verified by simulation. The nozzle is used on a precision motion control platform (shown in Figure 1), as a sub-part of the injection system. The nozzle types used in this study are based on commercial nozzle designs. The nozzle is a conventional nozzle with a divergent inlet and a cylindrical exit cross-section (shown in Figure 2). This article mainly uses Standard k to simulate the erosion status of nozzle wear-ε Turbulence model. Among them, turbulent kinetic energy k and dissipation rate ε represented as [17,18,19,20]:

(1) $$\frac{\partial (\rho \kappa )}{\partial t}+\frac{\partial (\rho \kappa \mu_{i})}{\partial \chi_{i}}=\frac{\partial }{\partial \chi_{j}}[(\mu +\frac{\mu_{i}}{\sigma_{k}})\frac{\partial \kappa }{\partial \chi_{j}}]+G_{\kappa }+G_{b}-\rho \varepsilon -Y_{M}+S_{K}$$

(2) $$\frac{\partial (\rho \varepsilon )}{\partial t}+\frac{\partial (\rho \varepsilon \mu_{i})}{\partial x_{i}}=\frac{\partial }{\partial x_{j}}[(\mu +\frac{\mu_{t}}{\sigma_{\varepsilon }})\frac{\partial_{\varepsilon }}{\partial x_{j}}]+C_{1\varepsilon }\frac{\varepsilon }{\kappa }(G_{k}+C_{3\varepsilon }G_{b})-C_{2\varepsilon }\rho \frac{\varepsilon^{2}}{\kappa }+S_{\varepsilon }$$

where the items in the equation are as follows:

*C*_1*ε*_, *C*_2*ε*_, *C*_3*ε*_—The empirical constant in the *k*-*ε* model, where *C*_1*ε*_ is 1.44, *C*_2*ε*_ is 1.92, and *C*_3*ε*_ is 0.09;
*∂_κ_*, *∂_ε_*—the corresponding Prandtl number in *k* and *ε*, where *∂_κ_* is 1.0 and *∂_ε_* is 1.3;
*S_k_*,*S_ε_*—user defined source items;
*μ_t_*—turbulent eddy viscosity coefficient;
*U_i_*—turbulence coefficient at coordinate *i*;
*X_i_*—coordinate component;
*G_b_*—generation term of k due to buoyancy;
*Y_M_*—pulsation expansion term in compressible turbulence;
*G_k_*—stress source term caused by velocity gradient.

### 2.2. Discrete Phase Model and Solution Condition Settings

**Figure 3:**
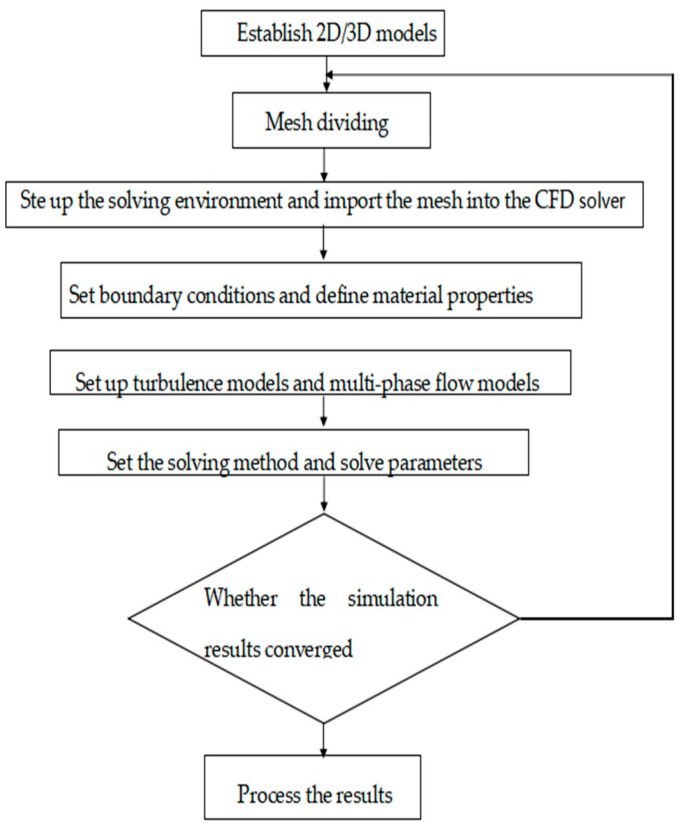
Flow chart of nozzle wear simulation.

A brief process of numerical simulation of cavity erosion situation of the nozzle based on Fluent is shown in Figure 3. Due to the complex turbulent motion of abrasive particles driven by water in the nozzle cavity and their high velocity, it is inevitable that abrasive particles will collide with the nozzle cavity. During the collision process, the momentum and energy of the abrasive change, and the lost momentum and energy, act on the wall of the nozzle cavity, causing nozzle erosion. The collisions and rebounds between the abrasive particles and the nozzle cavity are the main reasons for the wear on the nozzle cavity. Therefore, it is necessary to conduct a brief analysis of the collision between the abrasive particles and nozzle cavity surfaces. The process of collision and rebound of abrasives during movement can be simplified as tangential and normal motion. Based on the empirical formulas derived by Grant et al. [5] through extensive experiments, the normal and tangential rebound coefficients of solid particles at the wall can be obtained, and their expressions are as follows:

(3) $$e_{t}=\frac{v_{t_{2}}}{v_{t_{1}}}=0.998-0.029\theta +6.43\times 10^{-4}\theta^{2}-3.56\times 10^{-6}\theta^{3}$$

(4) $$e_{n}=\frac{v_{n_{2}}}{v_{n_{1}}}=0.993-0.0307\theta +4.75\times 10^{-4}\theta^{2}-2.61\times 10^{-6}\theta^{3}$$

where the items in the equation are defined as follows:

$v_{n1}$ and $v_{n2}$—the normal velocity of solid particles before and after impacting the wall surface;
$v_{t1}$, $v_{t2}$—tangential velocity of solid particles before and after impacting the wall;
*θ*—the angle between the trajectory of solid particles and the wall surface.

For the sake of generally analyzing the erosion of the nozzle cavity, the rebound of abrasive particles and empirical formulas for erosion to solve the corrosion rate should be considered at the same time. In general, the expression of the erosion empirical formula of the universal erosion model is as follows [21]:

(5) $$R=\sum_{n=1}^{N}\frac{m_{n}C\left(d_{n}\right)f\left(\theta \right){v_{n}}^{b\left(v\right)}}{A}$$

where *R* represents the corrosion rate. In Fluent numerical simulation, it is necessary to define the diameter coefficient $C\left(d_{n}\right)$, angle function $f\left(\theta \right)$, and velocity function $v_{n}$. Usually, in the angle function $f\left(\theta \right)$, when θ is 0°, 20°, 30°, 45°, and 90°, $f\left(\theta \right)$ values are 0, 0.8, 1.0, 0.5, and 0.4, respectively.

### 2.3. Mesh Generation

**Figure 4:**
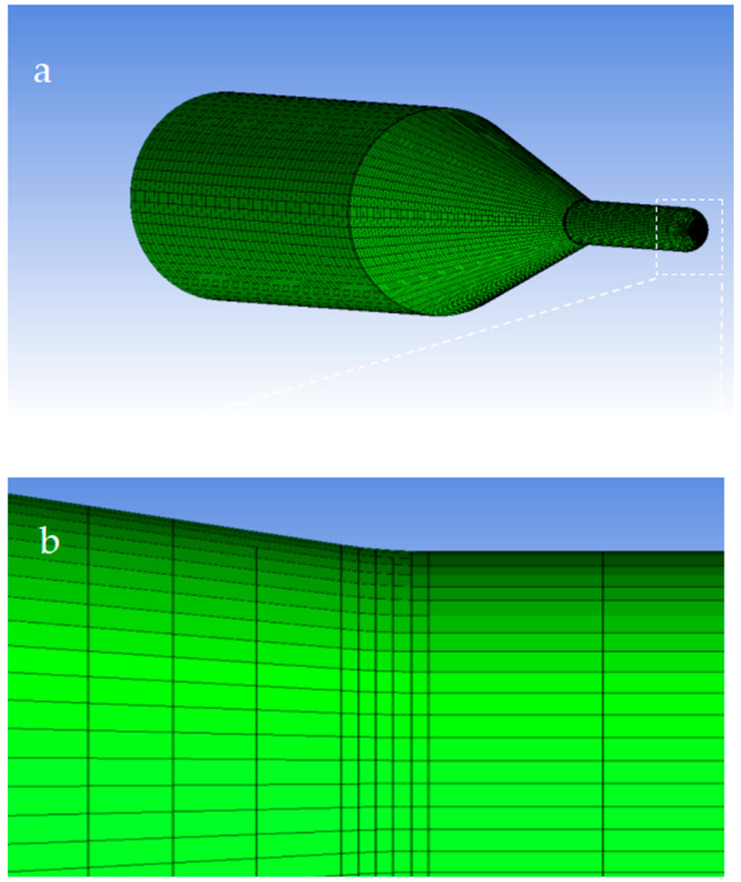
Mesh of nozzle geometry (**a**) and local magnification diagram (**b**).

**Figure 5:**
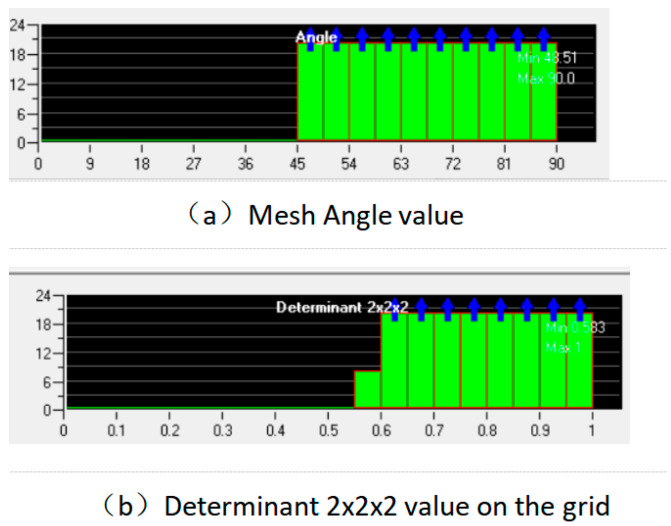
Grid quality check results.

The flow field in the nozzle cavity was meshed by using the meshing software ICEM CFD (Fluent 6.3.26). Firstly, the 3D model of the nozzle cavity flow field established in Space Claim is imported. In order to obtain more accurate results and make the numerical simulation solution converge better, the grid is encrypted at the nozzle inner diameter change and the details are shown in Figure 4. The nozzle geometry are meshed in Figure 4a. The number of hexahedral grids is 168,270, and the number of nodes is 174,264. In ICEM CFD, there is a determinant 2 × 2 × 2 on the angle, which is commonly used to judge the quality of the grid. The angle is between the edges of adjacent grids, ranging from 0 to 90°; 90° is the best grid quality, 0° is the worst. Generally speaking, when the angle is greater than 18°, it can meet the requirements for CFD calculation. Determinant 2 × 2 × 2 represents the Jacobi ratio, ranging from −1 to 1. When the value is 1, the grid quality is the best, and the smaller the grid quality is, the worse it is. When the value is less than 0, it means there is a negative-volume grid, which cannot be accepted by the CFD solver. The three-dimensional meshing quality of the nozzle is shown in Figure 5. The minimum value for the mesh Angle is 48.51, and most of the mesh angle values are above 72; The minimum determinant 2 × 2 × 2 value is 0.583, and most of the grid determinant 2 × 2 × 2 values are above 0.8. As can be seen from the above, the mesh quality is good, and there is no negative volume mesh, so it can be imported into Fluent for calculation solving.

## 3. Simulation Results of Nozzle Cavity Erosion

To study the erosion and distribution of different abrasive parameters in the nozzle cavity, this paper demonstrates three marked factors: abrasive water inlet pressure, abrasive particle diameter, and abrasive mass flow rate, on the erosion distribution and the erosion rate of the nozzle cavity. In actual working conditions, the inlet pressure of abrasive water, abrasive diameter, and abrasive mass flow rate should not be too large to polish an ultra-precision work piece. Some representative parameters are selected, and the related parameter details are shown in Table 1.

### 3.1. Effect of Different Abrasive Water Jet Inlet Pressures on Nozzle Cavity Wear

**Figure 6:**
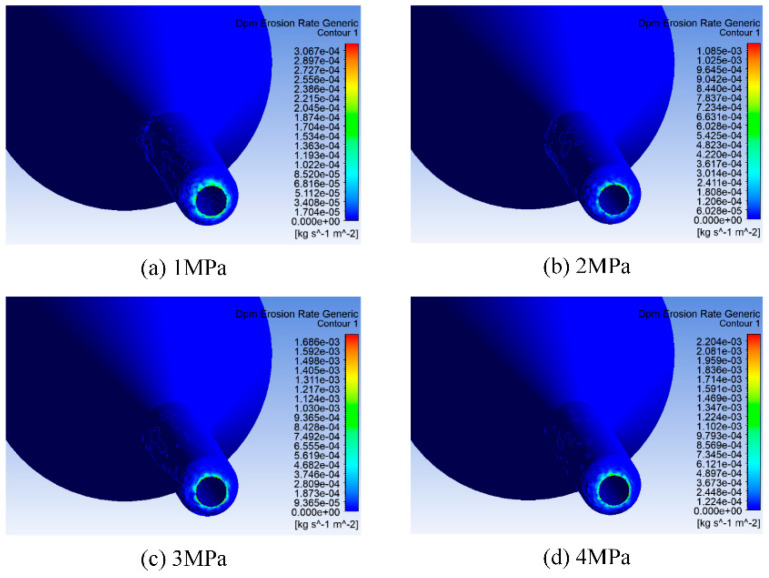
Cloud Chart of Erosion in the inner chamber of the nozzle under different jet pressures.

**Figure 7:**
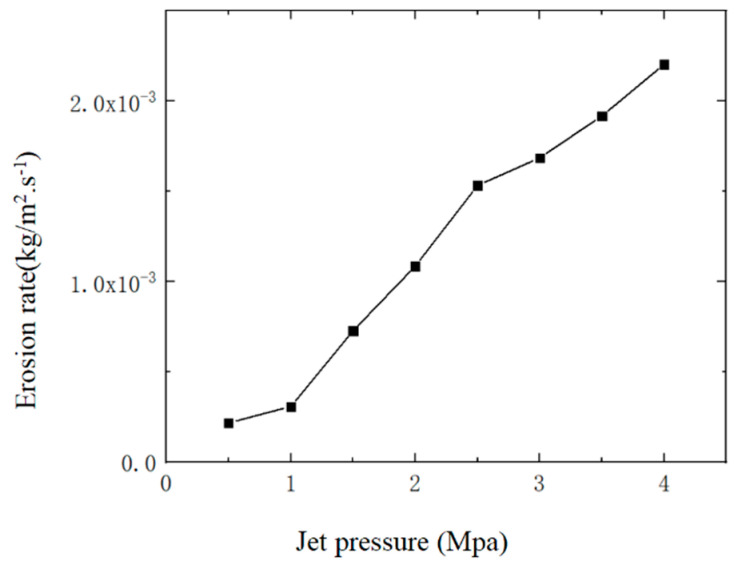
Changes in maximum erosion rate of nozzle inner cavity with different jet pressures.

The pressure change at the inlet of the fluid channel is set at 0.5–4 MPa according to the research needs, with a step size of 0.5 MPa. The outlet pressure is 0 MPa, the fluid partial density is 1 g/cm^3^, the dynamic viscosity is 1 Maps, and it is incompressible. The inlet pressure of the abrasive water jet determines the speed of abrasive and water at the nozzle inlet, and the speed of abrasive particles influence the impact force of the abrasive on the nozzle cavity, which ultimately affects the wear situation of the nozzle cavity. Therefore, the pressure of abrasive water at the nozzle inlet cannot be ignored. Numerical simulation was conducted on the wear of the nozzle cavity under different jet pressures. Eight values were taken within a jet pressure range from 0.5 MPa to 4 MPa, the specific values of which are shown in Table 1. The abrasive diameter and abrasive mass flow rate remain constant, with values of 1 um and 0.0005 kg/s, respectively. The erosion of the nozzle cavity is mainly concentrated at the nozzle outlet, so only four results from the nozzle wear distribution cloud map can be displayed to analyze the distribution of nozzle cavity wear. The cloud diagram of nozzle cavity wear under different jet pressures is shown in Figure 6 (e is the scientific notation, and e-x represents the negative x power of 10), and the relationship between the maximum erosion rate of the nozzle cavity and jet pressure is shown in Figure 7.

Under different jet pressures, the erosion of the nozzle cavity by abrasive particles is mainly concentrated at the nozzle outlet, seen in Figure 6. When the jet pressure is 1 MPa, the erosion of the abrasive on the nozzle outlet is relatively dispersed, and the abrasive also causes a certain erosion effect on the contraction section inside the nozzle. As the jet pressure increases, the erosion of the abrasive on the nozzle gradually concentrates at the nozzle outlet. At lower jet pressure, the abrasive moves at a lower speed inside the nozzle, stays inside the nozzle for a longer time, and the wear range caused by the collision and rebound of the abrasive in the nozzle cavity becomes larger. When the jet pressure increases, the speed of the abrasive driven by water also increases, and the residence time of the abrasive particles in the nozzle cavity decreases. Therefore, the scope of erosion is concentrated at the nozzle outlet. As the jet pressure increases, the maximum erosion of the nozzle cavity also increases, as seen in Figure 7. Therefore, under the premise of meeting the working conditions, appropriately reducing the jet pressure effectively improves the lifespan of the jet polishing nozzle.

### 3.2. Effect of Different Abrasive Diameters on Nozzle Cavity Wear

**Figure 8:**
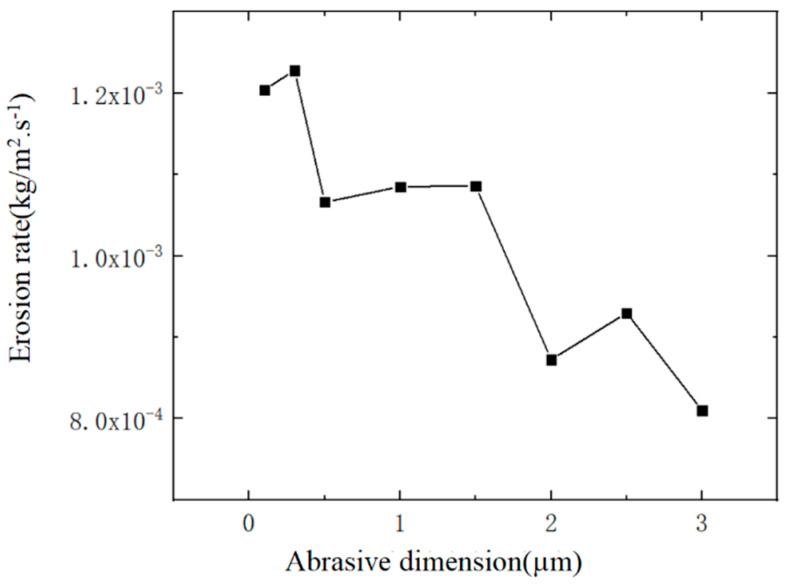
Variation of maximum erosion rate of nozzle inner cavity with abrasive diameter.

In the process of abrasive water jet polishing, the particle size of the abrasive can affect the removal rat of the work piece, so the change in abrasive diameter can also affect the wear of the nozzle cavity. Take 8 values within the abrasive diameter range from 0.1 um to 3 um, as shown in Table 1, and numerically simulate the wear of the nozzle cavity. The jet pressure and abrasive mass flow rate remain unchanged, taking 2 MPa and 0.0005 kg/s, respectively. Four results from the nozzle wear distribution cloud map are presented to analyze the impact of changes in abrasive diameter on the distribution of erosion wear in the nozzle cavity. The wear cloud map of the nozzle cavity under different abrasive diameters obtained through numerical simulation is shown in Figure 6, and the variation of the maximum erosion rate of the nozzle cavity with different abrasive diameters is shown in Figure 8.

As the diameter of the abrasive increases in Figure 6, the distribution of erosion in the nozzle cavity has a certain change, but the change is not significant, and the distribution of erosion is mostly concentrated at the nozzle outlet. From the curve in Figure 8, within the range from 0.1 um to 0.3 um in diameter, the maximum erosion rate of the abrasive on the nozzle cavity decreases with the increase of the abrasive diameter. The reason is that an increase in the diameter of the abrasive can increase the quality of the abrasive, and the kinetic energy obtained at the same jet speed is relatively large. The maximum erosion rate of the inner chamber should also increase.

### 3.3. Effect of Different Abrasive Mass Flow Rates on Nozzle Cavity Wear

**Figure 9:**
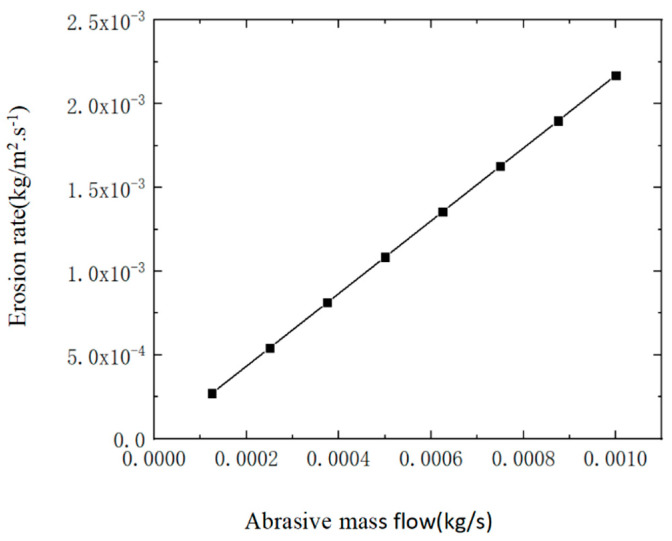
Variation of maximum erosion rate of nozzle inner cavity with abrasive mass flow rate.

By varying the mass flow rate and the shape factor of the abrasive particles, the velocities of the water jet flow, air and abrasive, and the erosion inside the focusing tube were studied. The paper defines the abrasive water jet pressure and abrasive diameter at the nozzle inlet, and the mass of abrasive particles in liquid is also an important factor causing wear in the nozzle cavity. Take 8 values from the abrasive mass flow rate of 0.000125 kg/s to 0.001 kg/s, as shown in Table 1, and conduct numerical simulation on the erosion of the nozzle cavity. The jet pressure and abrasive diameter remain unchanged, taking 2 MPa and 1 um, respectively. Four results of the nozzle erosion and wear distribution cloud map are presented here to analyze the impact of changes in abrasive mass flow rate on the erosion and wear distribution in the nozzle cavity. The cloud map of erosion distribution in the nozzle cavity under different abrasive mass flow rates is shown in Figure 6, and the variation of the maximum erosion rate in the nozzle cavity with different abrasive mass flow rates is shown in Figure 9.

It shows that when the abrasive mass flow rate ranges from 0.000125 kg/s to 0.001 kg/s in Figure 6, the changes in abrasive mass flow rate hardly affect the erosion distribution in the nozzle cavity. When the abrasive particles move inside the nozzle without changing the jet pressure, the mass flow rate of the abrasive is only related to its density in water, which is the concentration of the abrasive. Therefore, increasing the mass flow rate of the abrasive will increase the number of abrasive particles, on the premise that the jet pressure and abrasive diameter remain unchanged; changing the mass of abrasive particles in liquid only affects the number of abrasive particles and does not affect the movement trajectory of the abrasive. As the abrasive mass flow rate increases in Figure 9, the maximum erosion rate of the nozzle cavity also increases, and the change is significant. The reason is that the increase in the number of abrasive particles inside the nozzle intensifies the wear and corrosion of the nozzle cavity. Therefore, the abrasive mass flow rate should be minimized as much as possible to extend the lifespan of the jet polishing nozzle.

## 4. Numerical Simulation Analysis of Structural Changes in Nozzle Wear

Firstly, it is necessary to define the properties of the fluid and abrasive inside the nozzle cavity. Set the continuous medium material inside the nozzle cavity as water, and use the values provided in the Fluent database for density and viscosity. The discrete phase medium material remains at default, with a density of 3500 kg/m^3^. The flow density is set to 998.2 kg/m^3^, and the flow viscosity is set to 0.001 kg/m·s^−1^. The Standard model should be selected as the turbulence model, and the discrete phase model should be selected for the multiphase flow model. First, the interaction between discrete phase and continuous phase is turned on, and the Erosion/Accretion option is turned on in the Physical Models section, and the parameters of the particles are set in the Injection source. The solution method is the SIMPLEC algorithm the spatial discrete options and solution control remain unchanged by default, the residual standard is set to meet the accuracy, and iterative calculation can be started after initialization. After the calculation is finished, the calculation results will be exported to the post-processing software (Fluent 6.3.26.) CFD-Post for post-processing. The basic parameters of material properties are shown in Table 2.

**Figure 10:**
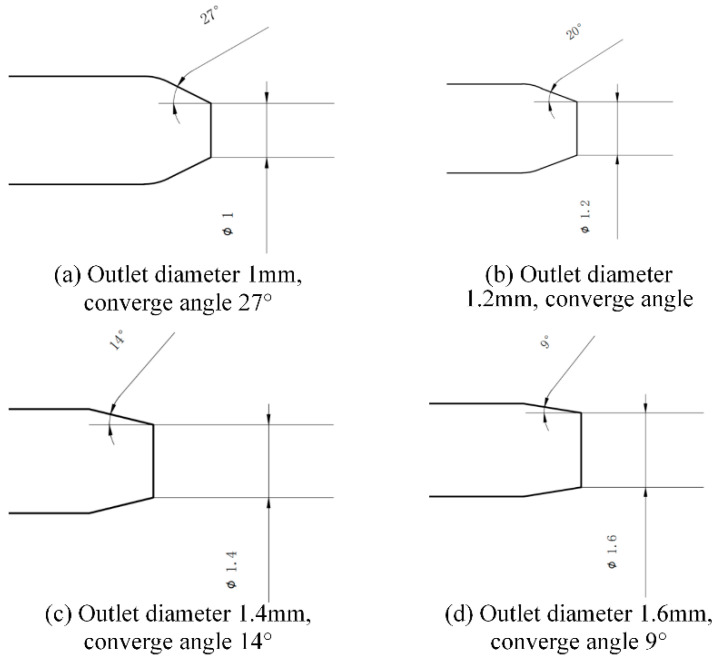
Geometric parameters of nozzle outlet under four different wear conditions.

It is known that the original diameter of nozzle is 1 mm, and the related convergence angle at the outlet is 27. The corresponding diameter after nozzle erosion is 1.2 mm, 1.4 mm, 1.6 mm, and the corresponding contraction angle values at the outlet are 20°, 14°, and 9°, which are shown in Figure 10. The geometric structure parameters before and after nozzle wear are shown in Table 3.

### 4.1. Numerical Simulation Solution Process

Reset the material properties of the abrasive to a density of 3500 kg/m^3^ and a viscosity of 0.0001 kg·s/m. The boundary conditions are set as pressure inlet and outlet, respectively. The inlet pressure is set to 2 MPa, and the volume fraction of abrasive at the inlet is set to 0.1. The outlet pressure is set to 0 MPa, and the operating conditions are set in the atmospheric environment; wall1 is the nozzle wall surface, wall2 is the work piece wall surface, and they are respectively set as non-slip wall conditions.

Next, set and initialize the solution method. Set the solution method as Coupled, keep the default values for other parameters, and set the residual to 10–6 to meet the accuracy requirements. When initializing the settings, first set the abrasive volume fraction in the entire flow field area all zone to 0.

Keep other parameters unchanged, and locally initialize the internal flow field of the nozzle. Set the abrasive volume fraction of the internal flow field of the nozzle to 0.1. After initialization is completed, set the number of solving steps to 2000, start numerical simulation and calculation, and the calculation ends at around 1200 steps.

### 4.2. Analysis of Jet Velocity in the Internal and External Flow Fields of Nozzles with Different Degrees of Wear

The paper predicts the geometric structure changes of nozzles under different degrees of wear and uses numerical simulation methods to analyze the internal and external flow fields of four nozzles with different degrees of wear, to study the impact of nozzles with different degrees of wear on jet performance.

**Figure 11:**
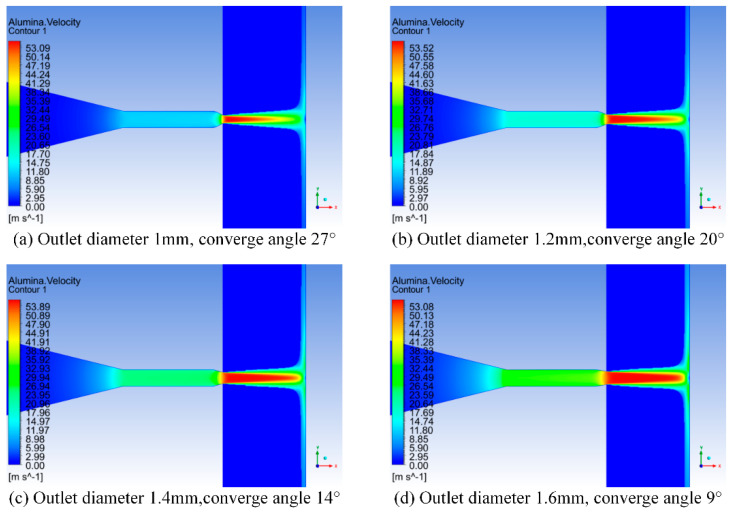
Cloud chart of abrasive velocity distribution for nozzles with different wear degrees.

**Figure 12:**
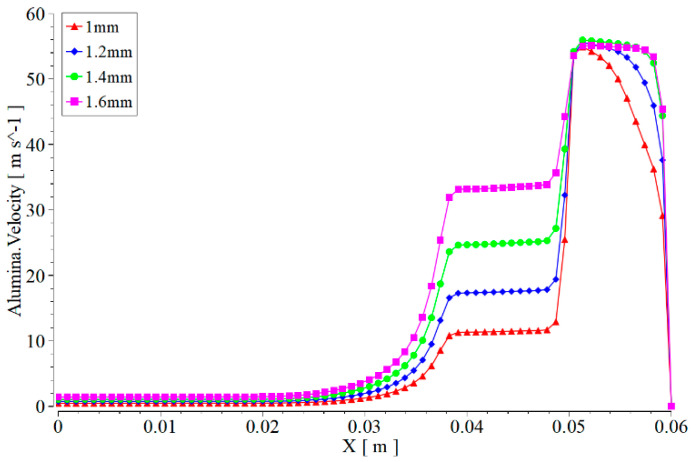
Variation of abrasive velocity of nozzles with different wear levels along the axis.

Regardless of the degree of wear of the nozzle in Figure 11, the jet structure of the external flow field of the nozzle is consistent with the theoretical jet structure analyzed above. The red areas with higher velocities in the cloud image are the iso-kinetic nuclei of the jet. Comparing the velocity cloud maps of the flow field inside and outside the nozzle with different degrees of wear, it can be seen that the greater the wear at the nozzle outlet(Figure 11), the longer the constant velocity core area of the jet is. At the same target distance, the constant velocity core of the jet is closer to the work piece; in addition, the water velocity at the contraction section before the nozzle outlet also increases with the increase of wear degree. To facilitate the analysis and comparison of the changes in nozzle jet velocity with different degrees of wear, the nozzle axis direction is taken as the X-axis, and the velocity of the water phase jet is taken as the Y-axis. The velocity variation of the water phase velocity along the nozzle axis direction is shown in Figure 12.

Due to the existence of two convergent angles inside the nozzle, seen in Figure 12, regardless of the degree of wear of the nozzle, the fluid inside the nozzle undergoes two accelerations. However, the nozzle with lower wear only reaches a velocity of about 10 m/s after the first acceleration of the fluid. As the degree of wear at the nozzle outlet increases, the velocity of the fluid after the first acceleration in the nozzle continuously increases. When the diameter of the nozzle outlet increases to 1.6 mm, The velocity of the fluid after the first acceleration will have reached around 30 m/s. The acceleration of the fluid at the second contraction angle inside the nozzle is opposite to that of the first contraction segment. The higher the wear of the nozzle at the outlet, the larger the outlet diameter, and the smaller the velocity change at the outlet. From the analysis, when the speed changes significantly at a certain location, the wear situation at that location is more severe. Therefore, as the wear at the nozzle outlet increases, the wear of the nozzle gradually shifts from the contraction section at the outlet to another contraction section inside the nozzle.

When fluid is ejected from the inside of the nozzle, the maximum velocity of the fluid is basically the same regardless of the degree of wear on the nozzle. However, the higher the degree of wear at the nozzle outlet, the longer the duration of the maximum velocity of the fluid ejected from the nozzle in the external flow field will be. This conclusion corresponds to the change in the degree of wear on the nozzle and the size of the constant velocity nucleus of the jet in Figure 11. When the fluid passes through the same target distance and acts on the work piece, the jet velocity of the nozzle at the nozzle outlet that does not generate wear is relatively small. However, as the wear degree at the nozzle outlet increases, the jet velocity acting on the work piece also increases, and the impact force on the work piece increases. Especially when the nozzle outlet diameter is worn to 1.4 mm or larger, the jet basically contacts the work piece at its highest speed.

This paper adopts the Euler multi-phase flow model to analyze the velocity cloud map and velocity variation of the abrasive phase, respectively. The cloud diagram of abrasive phase velocity in the internal and external flow fields of nozzles with different wear degrees is shown in Figure 10, and the variation of abrasive phase velocity along the axis of nozzles with different wear degrees is shown in Figure 11.

For the nozzle under different outlet wear conditions in Figure 11 and Figure 12, whether it is the velocity distribution cloud map of the abrasive or not, the conclusions obtained are still basically the same. It is worth noting that at the same speed, the impact and shear forces of the abrasive on the inner wall of the nozzle and the surface of the work piece are significantly higher than those of water.

### 4.3. Analysis of the Impact of Jet Flow from Nozzles with Different Degrees of Wear on the Work Piece

Analyze the impact of nozzles with different degrees of outlet wear on the surface of the work piece at the same standoff distance. Due to the impact of abrasive water on the surface of the work piece, which mainly includes the impact pressure of abrasive water on the surface of the work piece and the material removal caused by the shear effect of the abrasive, the two are analyzed separately.

**Figure 13:**
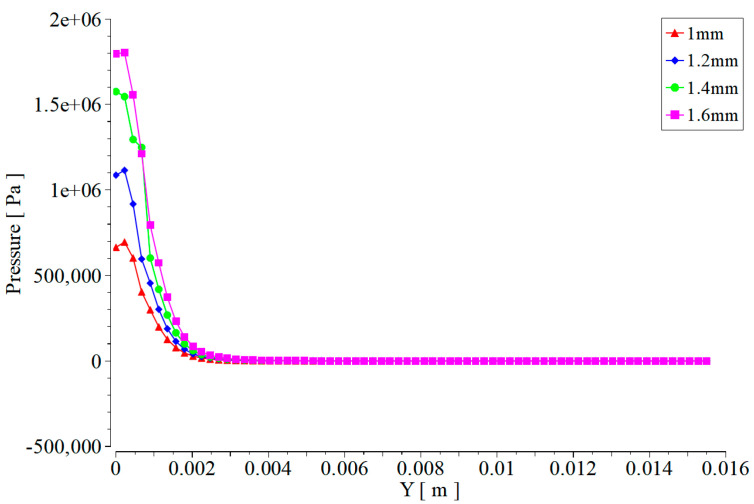
Pressure of nozzle jets with different degrees of wear on the surface of the work piece at the same target distance.

Taking the center of the work piece as the origin and the surface of the work piece as the X-axis, the pressure caused by abrasive water on the surface of the work piece is the Y-axis. The pressure caused by abrasive water jets with different degrees of wear at the nozzle outlet on the surface of the work piece is shown in Figure 13 (e is the scientific notation, and e-x represents the negative x power of 10).

**Figure 14:**
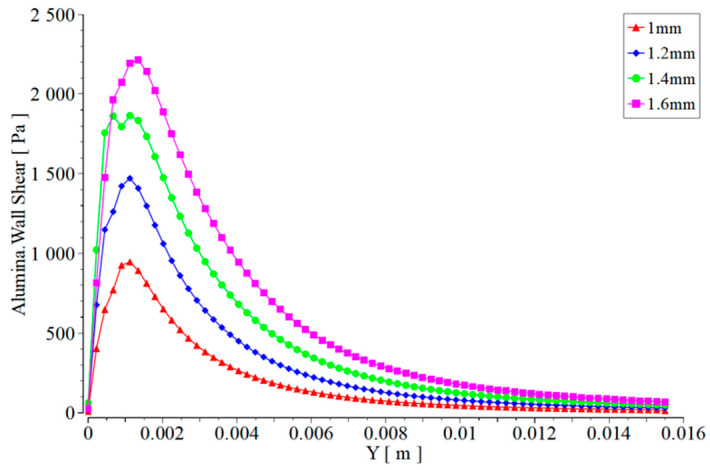
Lateral shear force of nozzle jets with different wear levels on the surface of the work piece at the same target distance.

As the degree of wear at the nozzle outlet increases, the pressure of abrasive water on the work piece also increases significantly, as seen in Figure 13. When the diameter of the outlet is worn to 1.6 mm, the pressure on the work piece caused by the abrasive water jet increases by more than double compared to the non-worn nozzle. The surface of the work piece is not only affected by the impact of jet pressure, but also by the lateral shear force caused by the jet. The transverse shear force of the abrasive water jet on the surface of the work piece is mainly caused by the impact motion of the abrasive, so only the transverse shear force of the abrasive on the work piece is shown here. Taking the center of the work piece as the origin and the surface of the work piece as the X-axis, the transverse shear force caused by the abrasive on the work piece surface is the Y-axis. The transverse shear force caused by the abrasive water jet at different nozzle outlet wear degrees on the work piece surface is shown in Figure 14.

It can be seen from Figure 14 that when the diameter of the nozzle outlet is worn to 1.6 mm, the shear force is 2.5 times higher than the shear force when the diameter is 1.0, which means that the jet force is divergent when the diameter is 1.6 mm, and the damage to the work piece is very serious.

## 5. Conclusions and Outlook

This study was based on Fluent to numerically simulate the internal and external flow fields of jet polishing nozzles. Four different types of nozzles with different wear degrees were modeled and numerically simulated to analyze the impact of nozzle structural changes caused by wear on the jet. The main conclusions of this article are summarized as follows:

1. The maximum erosion rate of the nozzle inner cavity is mainly affected by the jet pressure, abrasive particle diameter and abrasive mass flow rate. When the jet pressure and abrasive mass flow rate are higher, the maximum erosion rate caused by abrasive water on the nozzle inner cavity is greater. Abrasive diameter is inversely proportional to erosion rate.
2. Through the analysis of the velocity distribution at the nozzle axis, as the degree of wear at the nozzle outlet increases, the pressure of abrasive water on the work piece also increases significantly. When the diameter of the outlet is worn to 1.6 mm, the pressure on the work piece caused by the abrasive water jet increases by more than twice compared to the non-worn nozzle.
3. From the pressure distribution and transverse shear force distribution curves caused by the jet on the surface of the work piece, both the pressure caused by the jet on the work piece and the transverse shear force will increase with the increase of wear at the nozzle outlet. when the diameter of the nozzle outlet is worn to 1.6 mm, the shear force is 2.5 times higher than the shear force when the diameter is 1.0, which means that the jet force is divergent when the diameter is 1.6 mm, and the damage to the work piece is very serious. The increase of jet pressure and transverse shear force will have an impact on the processing quality of the work piece surface.

In order to facilitate model establishment and numerical simulation calculation, this article only predicts the structural changes of the inner cavity after nozzle wear has occured. However, in actual working conditions, the wear caused by abrasive on the inner cavity of the nozzle will exhibit irregular changes. The grooves and corrosion points caused by abrasive impacts are not reflected in the numerical simulation.

## Figures and Tables

**Table 1:** Simulation Parameters of abrasive water jet.

Inlet Pressure of Abrasive Water (MPa)	Abrasive Particle Diameter (μm)	Abrasive Mass Flow Rate (kg/s)
0.5	0.1	0.000125
1	0.3	0.00025
1.5	0.5	0.000375
2	1	0.0005
2.5	1.5	0.000625
3	2	0.00075
3.5	2.5	0.000875
4	3	0.001

**Table 2:** Basic parameters of material properties.

Current Density (kg/m^3^)	Water Flow Viscosity (kg/m·s^−1^)	Abrasive Grain Density (kg/m^3^)
998.2	0.001003	3500

**Table 3:** Geometry parameters and boundary conditions.

Parameters	Values Tested	Typical Value
Polishing pressure (MPa)	2	0.5–4
Abrasive flow rate (L/M)	4	3–5
Abrasive solid garnet	(#80) Garnet	(#80–#150)
Orifice diameter (mm) (before wear)	1	0.6–3
Orifice diameter (mm) (after wear)	1.2, 1.4, 1.6	N/A (not available)
Particle inlet diameter (mm)	3	3–5
Particle inlet position (mm)	Low, mid, high	N/A (not available)
Particle inlet angle (°)	0	0, 30, 45
Converging part angle (before wear)	27	N/A (not available)
Converging part angle (after wear)	20, 14, 9	N/A (not available)

## Data Availability

The raw data supporting the conclusion of this article will be made available by the authors, without undue reservation.
